# Epigenetics of hyper-responsiveness to allergen challenge following intrauterine growth retardation rat

**DOI:** 10.1186/s12931-014-0137-7

**Published:** 2014-11-13

**Authors:** Xue-Feng Xu, Qiong-Yao Hu, Ling-Fang Liang, Lei Wu, Wei-Zhong Gu, Li-Li Tang, Lin-Chen Fu, Li-Zhong Du

**Affiliations:** Department of Respiratory Medicine, The Children’s Hospital, Zhejiang University School of Medicine, Hangzhou, 310003 People’s Republic of China; Department of Neonatology, The Children’s Hospital, Zhejiang University School of Medicine, Hangzhou, 310003 People’s Republic of China; Department of Pediatric ICU, The Children’s Hospital, Zhejiang University School of Medicine, Hangzhou, 310003 People’s Republic of China; Department of Pathology, The Children’s Hospital, Zhejiang University School of Medicine, Hangzhou, 310003 People’s Republic of China

**Keywords:** Allergen, Asthma, Epigenetics, Endothelin-1, Intrauterine growth retardation

## Abstract

**Background:**

Epidemiological studies have revealed that intrauterine growth retardation (IUGR) or low birth weight is linked to the later development of asthma. Epigenetic regulatory mechanisms play an important role in the fetal origins of adult disease. However, little is known regarding the correlation between epigenetic regulation and the development of asthma following IUGR.

**Methods:**

An IUGR and ovalbumin (OVA)-sensitization/challenge rat model was used to study whether epigenetic mechanisms play a role in the development of asthma following IUGR.

**Results:**

Maternal nutrient restriction increased histone acetylation levels of the endothelin-1 (ET-1) gene promoter in lung tissue of offspring, but did not cause significant alterations of DNA methylation. The effect was maintained until 10 weeks after birth. Furthermore, these epigenetic changes may have induced IUGR individuals to be highly sensitive to OVA challenge later in life, resulting in more significant changes related to asthma.

**Conclusions:**

These findings suggest that epigenetic mechanisms might be closely associated with the development of asthma following IUGR, providing further insight for improved prevention of asthma induced by environmental factors.

## Introduction

An adverse intrauterine environment, such as that resulting from maternal malnutrition, may impact the development of the fetus resulting in fetal growth restriction or intrauterine growth retardation (IUGR) [1,2]. There is a wealth of epidemiological evidence to show that IUGR or lower birth weight is strongly correlated with an increased risk of adult diseases, such as type 2 diabetes mellitus, hypertension, and cardiovascular disease. This is called the “developmental origins of adult disease” theory or the “Barker” hypothesis [2]. Advances in perinatal or neonatal medicine have improved the survival of infants with IUGR, leading to an increase in the prevalence of adult diseases.

Studies have showed that IUGR or low birth weight was associated with medium and small airway obstruction, and altered lung function, including decreased forced expiratory volume in 1 s (FEV1), FEV1/forced vital capacity and forced mid-expiratory flow rate [3-5]. A number of studies have found a relationship between lower birth weight and asthma in children [6-8]. Furthermore, low birth weight predisposes an individual to develop asthma, and those who later become overweight adolescents have reduced lung function and the highest prevalence of asthma [9]. All respiratory function variables measured were significantly decreased in extremely low birth weight children compared with normal birth weight controls [10]. A recent meta-analysis showed that preterm birth and higher weight gain in infancy were closely associated with childhood asthma [11]. A study by Villamor *et al.* indicated that low birth weight was associated with a significantly greater risk of asthma independent of gestational age, sex, year of birth, or maternal age, parity, or socioeconomic status; among monozygotic twins, birth weight less than 2500 g was related to increased risks of asthma [12]. These investigations further revealed that, in addition to shared environmental or genetic factors, other regulatory mechanism such as DNA methylation or histone modifications might be involved in the development of asthma following either IUGR or low birth weight.

An increasing number of studies have showed that epigenetics plays an important role in the fetal origin of adult disease [13]. Epigenetics refers to all heritable changes in phenotype or gene expression states that are not involved in the DNA sequence itself. To date, epigenetic mechanisms in adult-onset diseases following IUGR, including type 2 diabetes mellitus, hypertension, and pulmonary arterial hypertension, have been extensively investigated [14-18]. However, whether epigenetics is involved in the pathogenesis of asthma following IUGR or low birth weight is not clear.

There is evidence that intrauterine nutrient (calorie) restriction may cause changes not only in placental DNA methylation [19], but may also affect epigenetic mechanisms in organs of the offspring. Our previous studies found that maternal nutrient restriction increased histone acetylation and hypoxia inducible factor-1α binding levels of the endothelin-1 (*ET-1*) gene promoter in pulmonary vascular endothelial cells from IUGR newborn rats, which continued up to 6 weeks after birth. These epigenetic changes resulted in an IUGR rat being highly sensitive to hypoxia later in life, causing more significant pulmonary arterial hypertension or pulmonary vascular remodeling [18,20]. A large number of studies have confirmed that asthma is an inflammatory airway disease characterized by bronchoconstriction and hyperreactivity with influx of inflammatory cells, mucus production, edema, and airway remodeling, while ET-1 has an important role in each of these processes [21,22]. Increased expression of ET-1 was strongly associated with the development of asthma [23-26]. Based on this evidence, we hypothesized that intrauterine nutrient restriction may not only cause epigenetic changes of the *ET-1* gene at the endothelial cellular level, but cause epigenetic alterations of the *ET-1* gene at a lung tissue level and persist into later life. Furthermore, we hypothesized that these epigenetic changes of lung tissue would induce an individual to be highly sensitive to allergens or other stimuli, resulting in more significant airway inflammation or asthma presentation.

In the present study, an IUGR rat model induced by maternal nutrient restriction was used to investigate the level of histone acetylation and DNA methylation of *ET-1* gene promoter region in lung tissues from 1-day-old and 10-week-old IUGR rats, and the reactivity of IUGR rats to ovalbumin (OVA) allergen challenge was assessed.

## Methods

### Intrauterine growth retardation (IUGR) rat model

**Figure 1:**
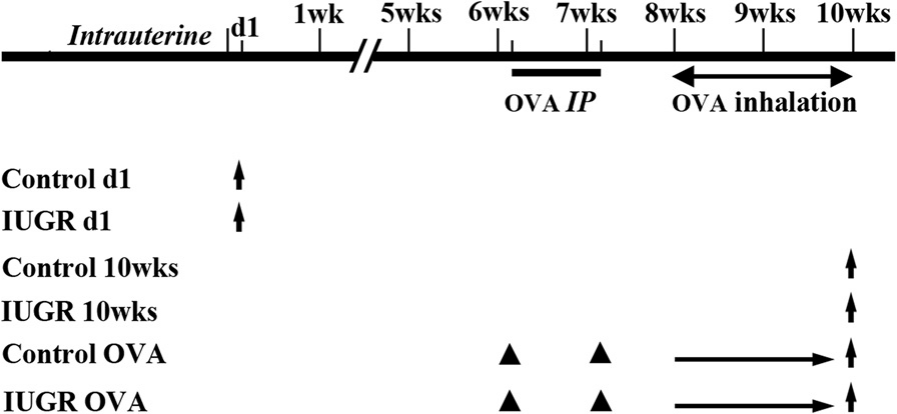
**Sampling age of different experimental group rats.** Upward arrows represent sampling times, solid triangles represent the times of interventions, d1 represents 1 day after birth. OVA = ovalbumin; IP = intraperitoneal injections.

This study was carried out in strict accordance with the recommendations in the Guide for the Care and Use of Laboratory Animals of the National Institutes of Health. The protocol was approved by the Committee on the Ethics of Animal Experiments of Zhejiang University. All surgery was performed under sodium pentobarbital anesthesia, and all efforts were made to minimize suffering. The IUGR rat model was established based on our previous study [18]. Virgin female Sprague–Dawley rats weighing 250-300 g obtained from Zhejiang University Laboratory Animal Center were mated overnight. The pregnant rats were randomly divided into two nutritional groups: a control group, and an under-nutrition group. Pregnant rats in the control group were fed a standard commercial rat diet, while pregnant rats in the under-nutrition group were fed the same diet at 50% of the free intake throughout gestation until birth. Both groups of rats were kept in the same room and had free access to water. Those pups whose birth weight was below the 10th percentile of normal birth weight were defined as IUGR. The litter size was culled to five pups per litter to assure adequate nutrition until weaning. The newborn pups from the control group were considered as a normal birth weight group, referred to as “Control d1”, and those newborn pups from the under-nutrition group were referred to as “IUGR d1”. Newborn IUGR rats continued to be reared by diet-restricted mothers that received normal food intake through lactation, while the control pups were reared by control mothers. Both groups of rats were raised until 10 weeks of age. The sampling ages of the different experimental group rats are presented in Figure 1.

### Sensitization and antigen challenge (asthma) model

Six-week-old IUGR and control rats were respectively sensitized by an intraperitoneal injection of 100 mg of ovalbumin (OVA, Sigma, USA) together with 100 mg Al(OH)3 in 1 ml 0.9% NaCl on the 1st day of the 7th and 8th weeks. After this 2-week sensitization, different groups of rats (each group containing 8–10 animals) were exposed to aerosolized OVA for 2 weeks. The aerosol was generated with an ultrasonic nebulizer (PARI, Germany) and was introduced into an exposure chamber. The median aerosol particle size was 3.9 μm according to the manufacturer’s specifications. The concentration of OVA in the nebulizer was 10% (wt/vol), and the duration of exposure was 30 min. The control and IUGR rats with OVA exposure were referred to as “Control OVA” and “IUGR OVA”, respectively. Lung tissues of different age groups were obtained, frozen in liquid nitrogen and stored at −80°C for further study.

The serum IgE level and hematoxylin and eosin (H&E)-stained lung tissue were used to evaluate rat asthma model. Serum was obtained by lethal cardiac puncture in anesthetized rats and stored at −80°C for measurement of IgE by ELISA. In H&E-stained lung sections, the number of eosinophils per square millimeter in the peribronchial and perivascular tissue was assessed using Image Pro Plus software (Media Cybernetics). More than eight bronchioles and pulmonary arteriole in a minimum of 10 high-power fields per lung tissue were randomly examined in a blinded fashion [27].

### Western blot detection for endothelin-1 protein

Total protein extracts were prepared and protein concentrations measured according to our previous studies [28,29]. Total protein extracts were prepared from lung tissues in a RIPA lysis buffer (50 mM Tris, 150 mM NaCl, 1% NP-40, 0.5% sodium deoxycholate, 0.1% SDS) containing protease inhibitors. Protein concentrations were measured using a BCA assay method. Thirty microgram protein extracts were resolved on 8-10% SDS polyacrylamide gels for ET-1 (21 kDa) proteins. Proteins were transferred onto a polyvinylidene fluoride membrane using a BioRad gel blotting apparatus. Membranes were incubated with a primary antibody against ET-1 (ab2786, abcam, 1:300 in antibody diluent) overnight at 4°C and with a peroxidase-conjugated secondary antibody for 60 min. To confirm equivalent sample loading, β-actin was used as an internal control. The bands of Western blots were quantitated by densitometry and normalized to β-actin using Image Pro Plus software (Media Cybernetics).

### Lung tissue histological and immunohistochemistry assessment

Lung tissue was fixed in formaldehyde solution and embedded in paraffin. The tissue was sectioned at a thickness of 4–5 μm, and stained with H&E solution for analyzing the difference between eosinophils [29]. For immunohistochemistry, the paraffin sections were rehydrated and transferred to 100% methanol with 3% hydrogen peroxide to eliminate endogenous peroxidase activity. Sections were then rinsed with PBS, and blocked with blocking serum before incubation with a primary antibody against ET-1 (ab2786, abcam, 1:250 in antibody diluent) overnight. Control sections were incubated with PBS instead of the primary antibody. A systematic sampling method was used to evaluate random, non-overlapping calibrated fields for each variable, and the terminal bronchioles were used as the independent landmark for selecting a small pulmonary arteriole [29,30]. We used Image Pro Plus software (Media Cybernetics) to make the measurements. Tissue sections were analyzed by one without knowledge of the group from which the tissue was taken. At least eight slides from each lung were taken and analyzed.

### Quantitative real-time PCR for ET-1

Total RNA was isolated from lung tissues according to the RNeasy protocol (Axygen). RNA was reverse-transcribed to cDNA using a reverse transcriptase kit (Takara) according to manufacturer’s protocols. Reverse-transcribed PCR analysis was performed using 37°C for 15 min, followed by 85°C for 5 s. Real-time quantitative PCR was performed using the ABI Prism 7500 Instrument following the Takara protocol. PCR was carried out in triplicate from each fraction using 94°C for 3 min, followed by 40 cycles of 94°C for 15 s and 60°C for 1 min. β-Actin was used as an internal control. Primer sequences for ET-1 (NCBI Reference Sequence: NM_012548.2) and β-actin are as follows: forward: 5’-aagcagacaaagaactccgag-3’, reverse: 5’-cgctttcaactttgcaactcg-3’; forward: 5’-gccaaccgtgaaaagatg-3’, reverse: 5’-tgccagtggtacgaccag-3’, respectively.

### Bisulfite genomic DNA sequencing

Bisulfite genomic DNA sequencing was performed as described in our previous study [28]. Genomic DNA was extracted from lung tissue and treated with 3 M sodium bisulfite as described previously. Briefly, DNA was denatured with 2 M NaOH and then treated with 10 mM hydroquinone and 3 M sodium bisulfite. After purification using the Wizard SV Plus kit column, the DNA was treated with 3 M NaOH and precipitated with 3 volumes of 100% ethanol, one-third volume of 10 M NH4OAc, and 2 μL of glycogen. The chemically modified DNA was then subjected to PCR. PCR was amplified using an ET-1 promoter-specific bisulfate primer (5’-agatttttaaaagttagaggygattaga-3’, and 5’-tctaatcrcctctaacttttaaaaatct-3’). Reactions were performed in a 25-μL volume containing 0.2 μM dNTPs, 10 mM Tris–HCl, 50 mM KCl, 2 mM MgCl2, 0.2 μM primer and 0.75 units of AmpliTaq Gold. PCR products were purified and cloned. The cloned PCR fragments from each sample were sequenced with M forward primer and a PRISM AmpliTaq DNA Polymerase FS Ready Reaction Dye Terminater Sequencing kit. Re-amplified DNA fragments were purified with Centri-Sep Columns and sequenced with an ABI PRISM 310 Genetic Analyzer.

### Chromatin immunoprecipitation assay

Chromatin immunoprecipitation (ChIP) assay was performed as described in a previous study [28]. A 100 mg sample of lung tissue was ground in liquid nitrogen, and fixed with formaldehyde. The cell pellets were suspended in lysis buffer and centrifuged, and the supernatants were discarded. The pellets were resuspended in SDS lysis buffer, sonicated, centrifuged and aliquots of soluble chromatin were collected. Aliquots of the supernatants were retained for representing the input chromatin. Aliquots were incubated overnight at 4°C with one of the following antibodies: 5 μL anti-acetylaled Histone H3 (06–599, Millipore), 5 μL anti-acetylated Histone H3 (Lys9) (H3K9, 07–352, Millipore), or 5 μL anti-acetylaled Histone H4 (06–866, Millipore). The immune complexes were precipitated with Protein A beads and then eluted. The input, unbound and bound fractions were incubated with 5 M NaCl to reverse crosslink. The DNA fragments were purified using the Qiaquick PCR Purification kit (Qiagen). The DNA fragments containing ET-1 site-specific sequences were quantified using real-time PCR. Relative quantification of PCR products were based on value differences between the bound and input using the ^△△^Ct method. The PCR primers for the ET-1 gene promoter were as follows: ET-1 promoter A1, F5’-ttgcctgtgggtgactaatc-3’, R5’-ccttcaccggagcgaaag-3’ (−197 to +25); ET-1 promoter A2, F5’-cctcttgattcttgaactctggg-3’, R5’-attagtcacccacaggcaac-3’ (−397 to −179).

### Statistic analysis

Values are expressed as mean ± SEM (standard error of mean). The continuous variables in the different groups were compared by one-way analysis of variance. Multiple comparisons were performed using the Student–Newman–Keuls test. A difference of P <0.05 was considered to be statistically significant.

## Results

### Establishment of rat IUGR OVA-exposed model

Our previous study showed that the 10th percentile for normal newborn rat birth weight was 5.87 g. Newborn rats weighing less than 5.80 g were considered to be IUGR [18]. The offspring rats from pregnant rats followed by diet restriction were in accordance with IUGR criteria. The average weight of 10-week IUGR rats was significantly lower than that of 10-week control rats (367.5 ± 5.4 g *vs* 389.3 ± 7.0 g, *P* = 0.027). The average weight of 14-week IUGR and Control rats were 479 ± 8.4 g and 457 ± 9.1 g, respectively; there was no statistical difference between them (*P* = 0.1). The IgE levels from Control and IUGR 10-week rats were 13.73 ± 1.07 and 14.95 ± 0.48 ng/mL respectively, and there was no statistical difference between them (*P* = 0.51). After OVA-sensitizing/challenging, the serum IgE levels were significantly increased compared with non-OVA-exposed rats (*P* <0.05). Furthermore, the serum IgE level in rats from the IUGR OVA group was higher than that in rats from the Control OVA group (26.20 ± 1.84 ng/mL and 22.09 ± 1.20 ng/mL respectively, *P* = 0.048).

**Figure 2:**
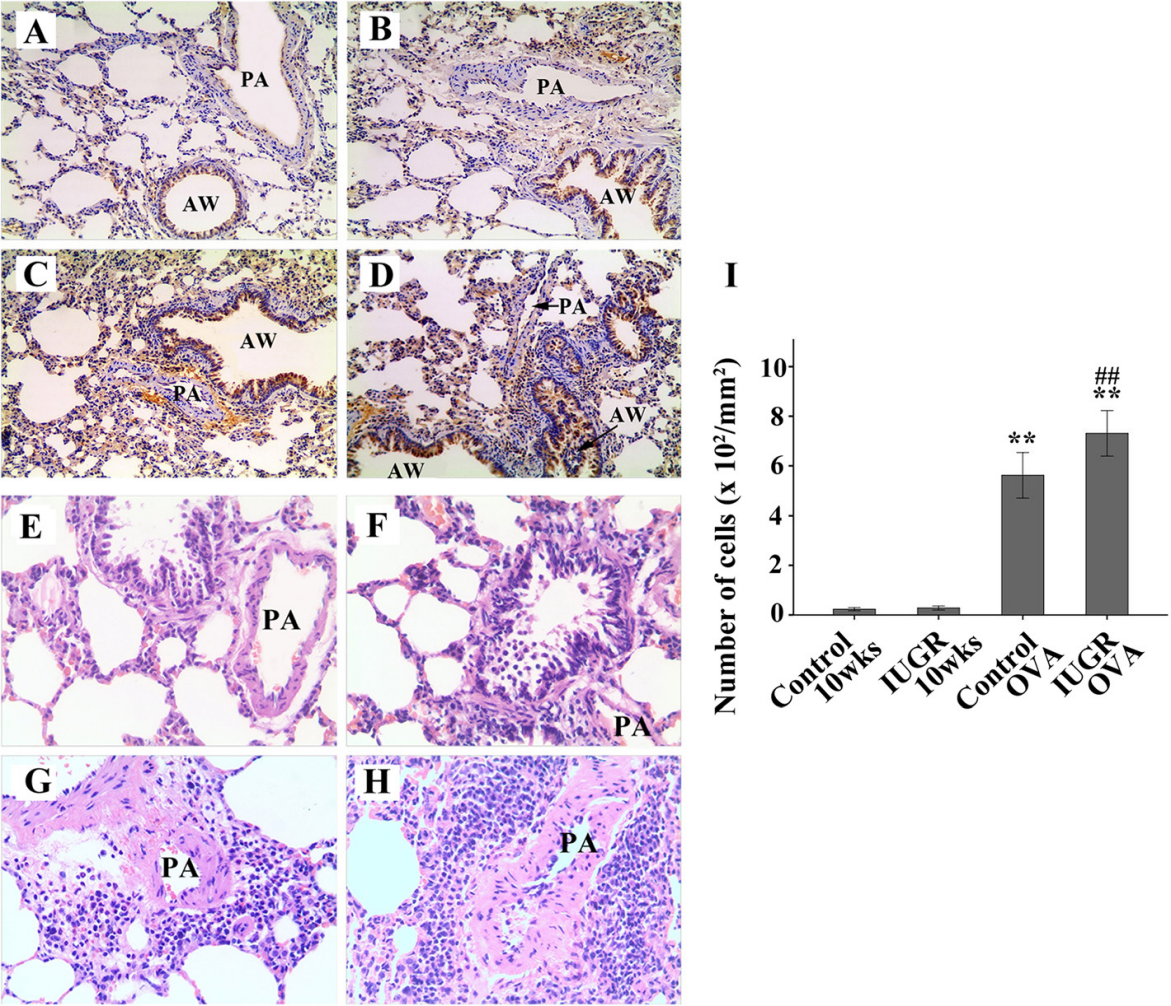
**Representative immunohistochemical staining for ET-1 (brown) and H&E staining in lung tissues from control 10 weeks (A, E), IUGR 10 weeks (B, F), Control OVA (C, G), and IUGR OVA (D, H) groups (×200).** The relative immunostaining densities for ET-1 from OVA-challenged groups were significantly higher than those from non-OVA-exposed groups, especially from IUGR OVA group. There were relatively few eosinophils infiltrations in the Control **(E)** and IUGR **(F)** rats. Tissue sections from the OVA-exposed IUGR **(H)** rats exhibited more predominant eosinophils infiltration compared with the OVA-exposed control rats **(G)** (×200). The histogram **(I)** showed the number of infiltrated eosinophil cells in peribronchiolar and perivascular lesions. **P <0.01 as compared with Control 10 weeks and IUGR 10 weeks groups, respectively; ##P = 0.001 as compared with Control OVA group. PA = pulmonary artery; AW = airway (n = 8).

A marked infiltration of inflammatory cells into peribronchiolar and perivascular lesions in the lung tissue sections from the OVA-exposed rats was observed compared with the non-OVA-exposed groups. Most of the infiltrating inflammatory cells were eosinophils. Tissue sections from the OVA-exposed IUGR rats exhibited more prominent eosinophil infiltration compared with the OVA-exposed Control rats. This phenomenon was observed mainly in the perivascular regions of the lung (Figure 2G, H).

### Lung tissue endothelin-1 (ET-1) levels and pathological changes

**Figure 3:**
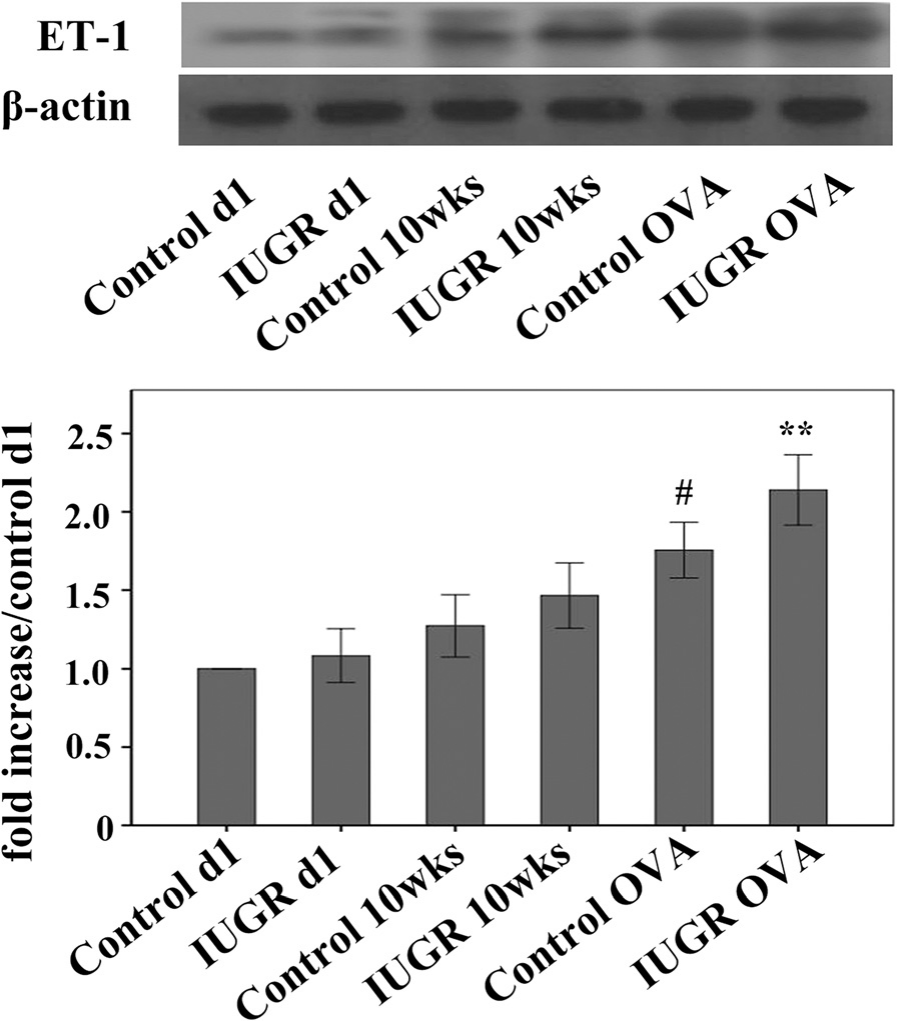
**Expression of lung tissue ET-1 proteins was measured using Western blot analysis.** The bar graph represents the relative ET-1 levels (percent of Control d1 ± SEM). ^#^P <0.05 as compared with Control d1, IUGR d1, Control 10 weeks, and IUGR 10 weeks groups, respectively; **P <0.05 as compared with Control d1, IUGR d1, Control 10 weeks, IUGR 10 weeks, and Control OVA groups, respectively; note increased ET-1 expression in the IUGR OVA group. β-actin protein expression served as an internal control and was used to normalize the protein band intensity (n = 4).

The ET-1 protein level of the IUGR d1 group was similar to that of the Control d1 group (*P* = 0.524). However, the ET-1 expression levels increased with age (Figure 3). Although the ET-1 protein level of the IUGR 10 wk group appeared to be higher than the age-matched control group, there was no statistically significant difference between them (*P* = 0.147). Lung tissue ET-1 protein expression levels in IUGR OVA and Control OVA-exposed groups were significantly higher than other non-OVA-exposed groups (*P* <0.05). Furthermore, the ET-1 level of the IUGR OVA group was significantly higher than that of the Control OVA group (*P* <0.01).

**Figure 4:**
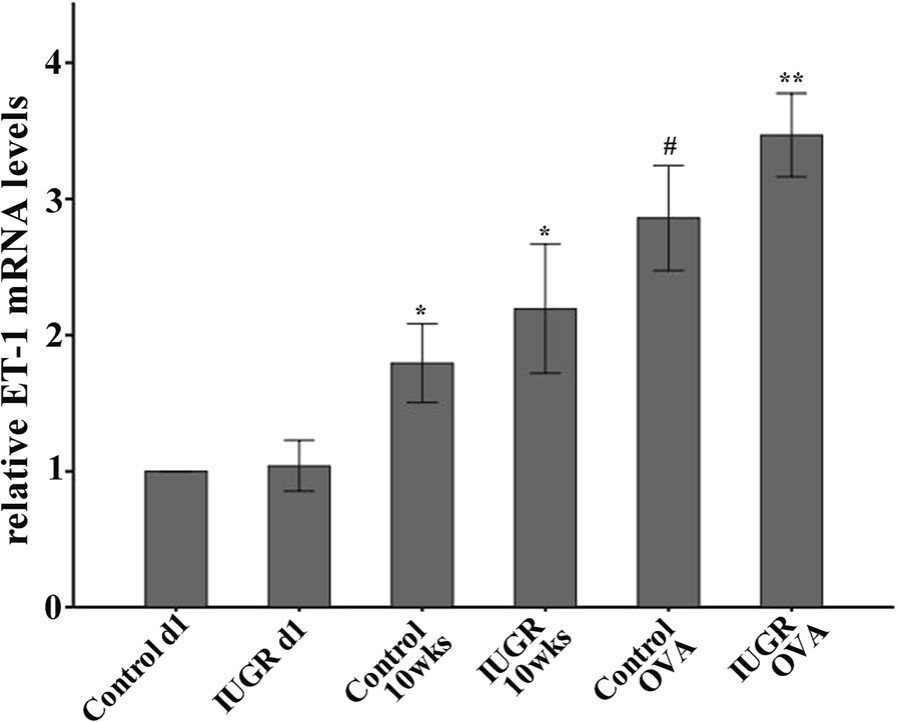
**Relative ET-1 mRNA levels were examined by quantitative real-time PCR.** Note that the ET-1 mRNA levels increased with age. *P <0.05 as compared with control d1, and IUGR d1, respectively; #P <0.05 as compared with control d1, IUGR d1, Control 10 weeks, and IUGR 10 weeks, respectively; **P <0.05 as compared with control d1, IUGR d1, Control 10 weeks, IUGR 10 weeks, and Control OVA group, respectively (n = 4).

Similar to protein expression patterns, the ET-1 mRNA levels increased with age (Figure 4). Lung tissue ET-1 mRNA levels in IUGR and Control OVA-exposed groups were significantly higher than in non-OVA-exposed groups (*P* <0.05). Moreover, the ET-1 mRNA level of the IUGR OVA group was significantly higher than that of Control OVA group (*P* = 0.017). These results indicate that the sensitivity to OVA in IUGR rats was significantly higher than in the normal control group.

Significant histological changes consistent with asthma were observed in lung tissues of the OVA-challenged rat models. These included increased ET-1 expression in the airway, and infiltration of inflammatory cells (Figure 2). ET-1 protein was mainly expressed in vascular endothelial cells and airway epithelial cells. In comparison with the age-matched control and IUGR rat groups, the number of airway epithelial cells expressing ET-1 was significantly increased in OVA-exposed animals, especially those in the IUGR OVA group (Figure 2D). Moreover, there was significant inflammatory cell infiltration in the lung tissue from IUGR OVA group (Figure 2H).

### Methylation status of CpGs in the ET-1 core promoter of lung tissue

**Figure 5:**
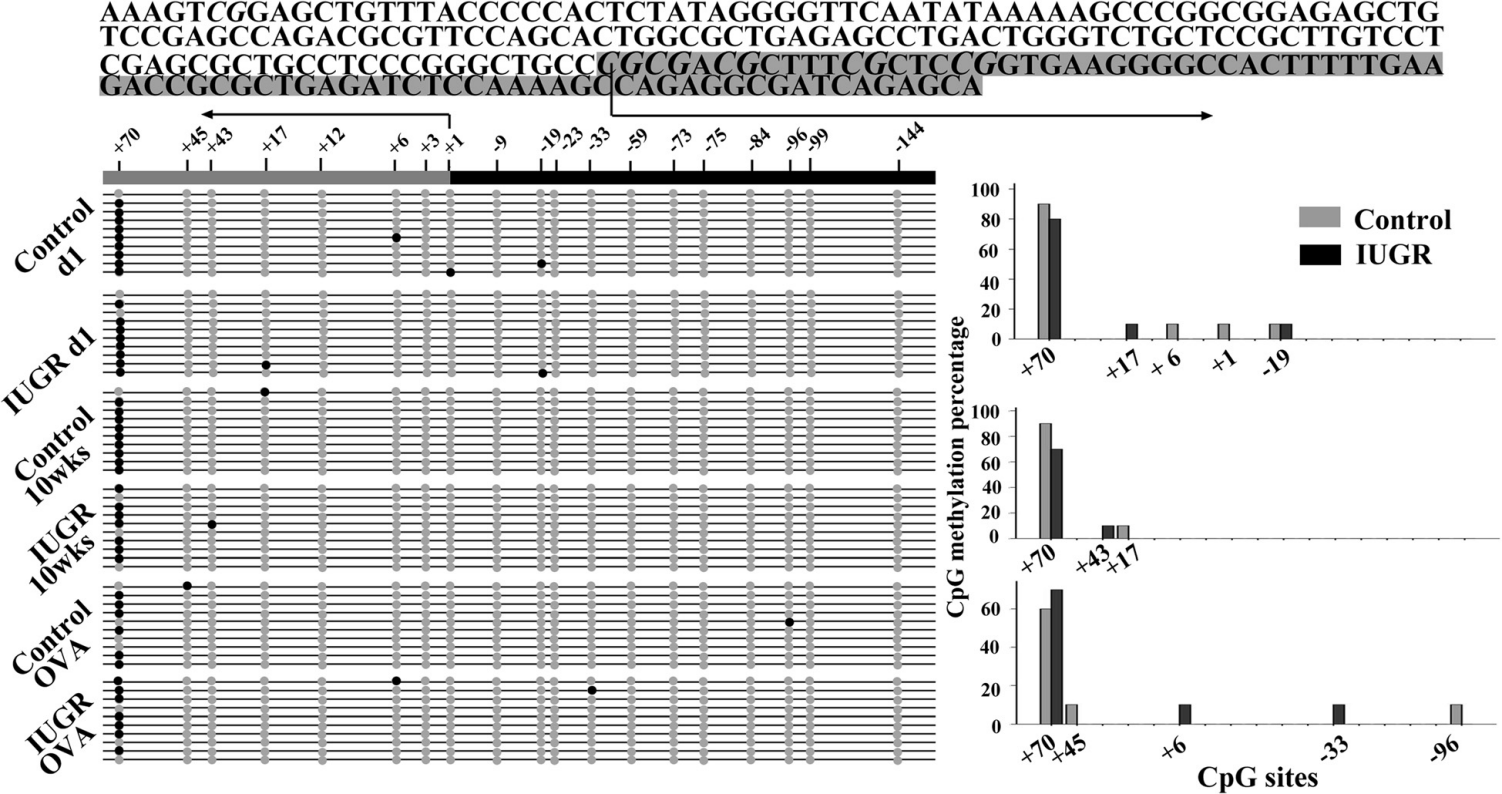
**Methylation profiles of the** ***ET-1*** **core promoter in lung tissue from IUGR and control rats are shown.** The proximal promoter sequence of *ET-1* (−149 to +80) is shown. Methylated CpG sites are indicated by solid black circles and unmethylated CpGs are depicted by grey circles. Arrows represent transcription start sites. Each line of circles represents independent PCR clones (n = 10). The bar graph represents CpG methylation percentage at each site, there was no significant difference between the Control and IUGR animals. Only the +70 site was hypermethylated, whereas sites +45, +43, +17, +6, +1, −19, −33, and −96 were hypomethylated, and the sites +12, +3, −9, −23, −59, −73, −75, −99, and −144 were unmethylated. There were no significantly statistical differences between Control and IUGR rats (P >0.05) at any of CpG sites.

Nineteen CpG sites within the proximal promoter of the rat *ET-1* gene were analyzed for methylation status. Representative results of bisulfate genomic DNA sequencing analysis are shown in Figure 5. Generally, 19 CpG doublets of the proximal promoter were lightly methylated in lung tissue. Only the +70 site was hypermethylated, whereas sites +45, +43, +17, +6, +1, −19, −33, and −96 were hypomethylated, and sites +12, +3, −9, −23, −59, −73, −75, −99, and −144 were unmethylated. At the +70 site, methylation rates were 80% and 90% in IUGR and Control d1 groups, 80% and 90% in the IUGR and Control 10-week groups, and 70% and 60% in the IUGR and Control OVA-exposed groups respectively, there were no statistically significant differences for any of these comparisons (P = 0.50). In other CpG sites, no significant differences were observed. These results show that intrauterine malnutrition was not sufficient to cause a significant change in DNA methylation of the *ET-1* gene.

### The ET-1 gene promoter histone code in lung tissue

**Figure 6:**
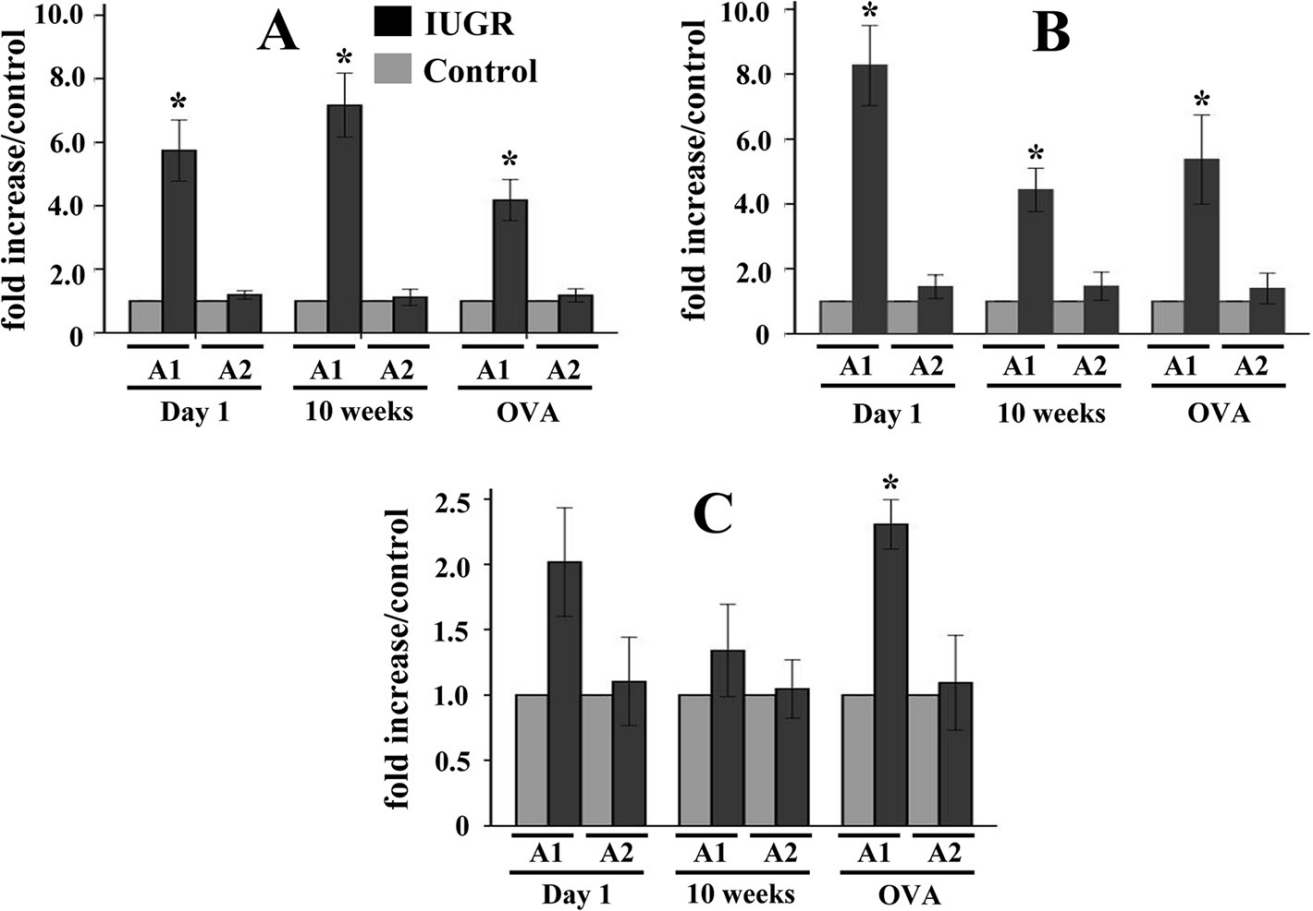
**Comparison of histone H3 (A), H3K9 (B), and H4 (C) acetylation levels at the** ***ET-1*** **promoter between IUGR and control rats using ChIP and relative quantitative real-time PCR.** A1 and A2 represent two areas of the *ET-1* gene promoter (−197 to +25, and −397 to −179, respectively). Data are expressed as IUGR percent of the Control ± SEM. *P <0.05 as compared with Control groups. Note that the levels of acetylated histone H3 and H3K9 in the *ET-1* promoter A1 region of lung tissue from IUGR groups were significantly higher than those from the age-matched Control groups; while in the *ET-1* promoter A2 region there was no significant statistical difference between IUGR and Control groups. The level of acetylated histone H4 in the *ET-1* promoter A1 region of the IUGR OVA group was significantly higher than that of the Control OVA group (n = 4).

Two sites in the *ET-1* gene promoter were analyzed for acetylated histone H3, H3K9, and H4. Acetylation levels of each site in the IUGR groups were quantified by ChIP/real-time PCR and are expressed as a fold-change relative to the same site in the age-matched Control groups. The levels of acetylated histone H3 in the *ET-1* promoter A1 region of lung tissue from IUGR d1, IUGR 10wks, and IUGR OVA groups were significantly higher than those from the age-matched Control groups (*P* = 0.039, 0.025, and 0.008, respectively). In the *ET-1* promoter A2 region, there was no statistically significant difference between IUGR and Control groups (*P* >0.05, Figure 6A). A trend of acetylated H3K9 levels in the *ET-1* promoter in lung tissue was similar to the observed change in acetylated histone H3 (Figure 6B).

Although the acetylated histone H4 levels in the *ET-1* promoter A1 region from IUGR d1 and 10-week groups were higher than those of the age-matched Control groups (Figure 6C), there was no statistical significance between them (*P* = 0.135, and 0.39, respectively). However, the level of acetylated histone H4 in the *ET-1* promoter A1 region of the IUGR OVA group was significantly (*P* = 0.02) higher than that of the Control OVA group. Similar to the changes of acetylated H3 and H3K9, there was no statistically significant difference in the changes of acetylated H4 in the *ET-1* promoter A2 region between IUGR and Control groups.

The ChIP experiments revealed that histone acetylation of the *ET-1* gene promoter might induce the higher sensitivity of IUGR rats to OVA, leading to the ET-1 protein expression and airway hyper-responsiveness.

## Discussion

The main finding of this study is that maternal nutrient restriction increased the histone acetylation levels in the *ET-1* gene promoter of lung tissue from IUGR newborn rats, but was insufficient to cause significant DNA methylation alterations. The effect could also be observed 10 weeks after birth. Furthermore, these epigenetic changes induced IUGR individuals to be highly sensitive to OVA challenge later in life, resulting in more significant changes related to asthma.

Epigenetics is the study of mitotically heritable changes in phenotype that occur without direct alterations of the DNA sequence itself. These epigenetic changes include DNA methylation, histone modification, and aberrant expression of microRNAs [31]. Generally, DNA methylation of a gene promoter region can directly repress transcription. Increased levels of histone acetylation are associated with increased transcriptional activity, whereas decreased levels of acetylation are closely correlated with suppressed gene expression [1]. Multiple epigenetic mechanisms are involved in regulation of asthma-related genes known to initiate and maintain the asthma phenotype and its symptoms [31]. There is evidence that exposure to adverse environmental factors during prenatal or postnatal life triggers the early airway to undergo a different course of development, resulting in a phenotype of increased sensitivity to allergens or irritants, hyperresponsiveness, and a skewed TH2 response. Exposure of pregnant mice to a diet rich in methyl donors increased the risk of allergic airway disease in the offspring through inducing methylation changes of asthma-related genes [32]. Maternal polycyclic aromatic hydrocarbons (PAH) exposure induced promoter methylation of an asthma related gene, IFN-γ, in cord white blood cells from children in a study cohort [33]. These findings demonstrate that dietary factors can alter asthma risk through epigenetic mechanisms during a susceptible period of developmental reprogramming. Our data demonstrate that maternal nutrient restriction can increase histone acetylation levels of the *ET-1* gene promoter in rat lung tissue. Increased histone acetylation at the *ET-1* gene promoter in IUGR rats is essential for transcription. Upregulated ET-1 protein expression in lung tissue from the IUGR asthma group rats is closely associated with the presence of increased acetylated histones. Reduction of these markers in Control asthma rats results in decreased ET-1 protein expression.

The lung has the highest levels of ET-1 secreted by endothelium, smooth muscle, and airway epithelium. In the airway, ET-1 is primarily localized to the bronchial smooth muscle with low expression in the epithelium [22]. ET-1 is a potent bronchoconstrictor, being approximately 100 times more potent than methacholine in asthma [21]. In a murine asthma model, ET-1 was shown to direct airway remodeling and hyper-reactivity [34]. The release of ET-1 from bronchial epithelium may play an important role in the increase of airway inflammation which was observed after postexercise bronchoconstriction in asthmatic patients [35]. These findings indicate that ET-1 is an important mediator in the development of asthma. Consistent with the regulation of the expression of many genes, ET-1 expression is also regulated by epigenetic mechanisms, and some transcription factor binding sites are present in the ET-1 proximal promoter region [36-38]. In the present study, IUGR rats with OVA exposure showed significantly increased ET-1 mRNA and protein levels in lung tissue. Furthermore, the immunohistochemical localization indicated that the number of airway epithelial cells expressing ET-1 significantly increased in OVA-exposed rats, especially those in the IUGR OVA group. These results further demonstrated that the increased ET-1 levels (including protein and mRNA levels) in lung tissues from OVA-exposed rats might be induced mainly by airway epithelial cells. The high ET-1 expression from IUGR OVA-exposed rats could be closely associated with increased histone acetylation within the *ET-1* gene promoter.

In our study, ChIP was performed to analyze the levels of histone acetylation in the *ET-1* gene promoter. The rat *ET-1* promoter contains consensus sequences that are required for transcription factor binding. Our results indicate that acetylation levels of histone H3 and histone H4 significantly increased in lung tissue from IUGR newborn rats compared with Controls. In the two *ET-1* promoter regions that we investigated, acetylation levels of histones within the proximal promoter region were higher in histone H3 or H4, although there was no significantly statistical difference in histone H4. In contrast, histone acetylation levels in the promoter region furthest from the transcription start site in IUGR rats were not significantly different compared with those in the Controls. These findings indicate that nutrient restriction in uterine may not only cause epigenetic alteration of the *ET-1* gene in pulmonary vascular endothelial cells, but may induce epigenetic alteration of the *ET-1* gene throughout the whole lung, probably including endothelial cells, airway epithelial cells, and smooth muscle cells. Furthermore, these epigenetic markers may persist until 10 weeks after birth, and induce an IUGR individual to be highly sensitive to allergen challenge later in life, resulting in more significant changes associated with asthma. In our previous study, persistent pulmonary hypertension of the newborn induced by short-term hypoxia and indomethacin treatments in uterine were not sufficient to cause significant CpG methylation in the endothelial nitric oxide synthase promoter. In the present study, maternal nutrient restriction was insufficient to induce significant methylation changes in the *ET-1* promoter. Whether the methylation status of CpG islands of other genes associated with asthma is involved in the underlying mechanism requires further research.

Epigenetic modifications of the *ET-1*gene in part explain the phenomenon of significant asthma-linked changes in IUGR OVA-exposed rats. This also further confirms the fetal origins of adult disease hypothesis and provides a potential mechanism of fetal programming. Because of the potential reversibility of epigenetic mechanisms, some appropriate interventions during the early stage of life may be likely to reduce the risk of adult disease through preventing or reversing the epigenetic modifications of the correlated genes. In addition to ET-1, there might be other factors involved in the development of asthma following IUGR. This would be worthy of further investigation. Nevertheless, caution is necessary when attempting to apply information from a rat model to human pathophysiology. Given the non-specificity of some interventions that influence epigenetic mechanisms, we did not analyze histone acetylation levels of the *ET-1* gene in lung tissue after such an intervention in the present study, and have not yet investigated the corresponding epigenetic changes in the next generation. Possible strategies for an early intervention and the potential identification of epigenetic biomarkers require further study.

## Conclusions

We found that maternal nutrient restriction increased histone acetylation levels of the *ET-1* gene promoter in lung tissue, but was insufficient to cause significant DNA methylation alterations. The effect persisted until 10 weeks after birth. These epigenetic changes may induce an IUGR individual to be highly sensitive to OVA (allergen) challenge later in life, resulting in more significant changes related to asthma. These findings suggest that epigenetics might be closely associated with the development of asthma following IUGR, providing further insight for improved prevention of asthma induced by environmental factors.
